# Uncontrollable Vomiting of Pregnancy

**Published:** 1907-07

**Authors:** 


### UNCONTROLLABLE VOMITING OF PREGNANCY.

Baisch reviews the experiences at Doderlein's clinic at Tubingen with twenty cases of uncontrollable vomiting in pregnant women. In three cases there was a history of chronic stomach trouble, and the vomiting was arrested in two by dietetic measures alone, restricting the patients to fluid foods. The third patient had become so debilitated that it was deemed dangerous to delay further and the pregnancy was interrupted. The vomiting ceased at once on removal of the ovum. In some cases of uncontrollable vomiting in previously healthy women, he states that there seems to be an overproduction in the ovum or uterus of some substance which induces vomiting. This uterine form of hypermesis gravidarum requires, besides the dietetic measures, some medication to reduce the excitability of the vomiting center. Drugs that can be given subcutaneously should be preferred. As morphin is eliminated through the stomach mucosa and is thus liable to add to the tendency to vomit, he prefers scopolamin; he injects from 0.3 to 0.5 mg. (1-200 to 1-128 grain) once or twice a day. This depresses reflex excitability and thus opens the way for effectual dietetic treatment. Stimulation of diuresis and evacuation of the intestines are ensured by repeated copious enemas. The majority of cases of uncontrollable vomiting are those of central origin, and these, he says, are the most easily influenced. Absolute bed rest is the principal measure, and the ordering of a thermophor to the stomach or ice bag to the abdomen is a plausible excuse and ensures quiet reclining on the back. All food and fluids are prohibited during the first twenty-four hours. Thirst is assuaged by transfusion of physiologic salt solution. The patient is then given milk by the teaspoonful, ice cold, and the next day a little zwieback, tea or coffee, gruel or bouillon. Fluid food should predominate and should be given in small quantities, not too often, and very gradually increasing the amounts allowed. The sensation of hunger is the first sure sign of commencing recovery. An abrupt change to ordinary food is liable to entail a discouraging relapse. If these measures fail, the patient must be removed at once to an institution where a hysteric tendency can be combated. Two of the 15 patients who belonged to this "central origin" group suffered from excessive salivation besides the incessant vomiting. Their condition soon became so grave that the pregnancy had to be arrested. The other 13 were cured by the rest and dietetic measures. The pregnancy was arrested in 5 of the total 20 cases of uncontrollable vomiting observed in an experience of 22,500 pregnant women during the last 5 years. It was deemed unwise to allow the women to go too far without radical relief. Those who are allowed to become so debilitated by the uncontrollable vomiting as to reach the brink of the grave before relief is given, are exposed later to a series of dangers, not the least of which are tuberculosis and polyneuritis to which they are unable to oppose any resistance. In case of induced abortion two colleagues should be called in consultation, and the uterus emptied at one sitting, after introducing a thick laminaria tent in the cervix the night before. The whole ovum can then be readily removed with forceps with little hemorrhage and the manipulations on the uterus reduced to the minimum. The patient can generally eat a substantial meal the evening of the same day.—Berlin Klin Woch, in Jour. A. M. A., June 1, '07.

